# Reactive Oxygen Species Generation in Human Cells by a Novel Magnetic Resonance Imaging Contrast Agent

**DOI:** 10.1155/2018/6362426

**Published:** 2018-03-26

**Authors:** Li Wang, Eric Lin, Mary J. Johansen, Timothy Madden, Edward Felix, Karen S. Martirosyan, Steven J. Frank

**Affiliations:** ^1^Department of Experimental Radiation Oncology, The University of Texas MD Anderson Cancer Center, Houston, TX, USA; ^2^Strategia Therapeutics, Inc., Boston, MA, USA; ^3^InPharma LLC, The Woodlands, TX, USA; ^4^Department of Experimental Therapeutics, The University of Texas MD Anderson Cancer Center, Houston, TX, USA; ^5^Department of Physics, University of Texas Rio Grande Valley, Rio Grande Valley, TX, USA; ^6^Department of Radiation Oncology, The University of Texas MD Anderson Cancer Center, Houston, TX, USA

## Abstract

The novel positive-contrast magnetic resonance imaging (MRI) marker C4 consists of an aqueous solution of cobalt chloride (CoCl_2_) complexed with the chelator N-acetylcysteine (NAC). We evaluated whether the presence of C4 or its components would produce reactive oxygen species (ROS, including hydroxyl, peroxyl, or other reactive oxygen species) in cultured cells. Human cancer or normal cells were incubated with 1% (w/v) CoCl_2_·6H_2_O or 2% NAC or a combination of both (1% CoCl_2_·6H_2_O : 2% NAC in an aqueous solution, abbreviated as Co : NAC) in the presence or absence of H_2_O_2_. Intracellular ROS levels were measured and quantified by change in relative fluorescence units. Student's *t*-tests were used. In all cell lines exposed to 1000 *μ*M H_2_O_2_, the Co : NAC led to ≥94.7% suppression of ROS at 5 minutes and completely suppressed ROS at 60 and 90 minutes; NAC suppressed ROS by ≥76.6% at 5 minutes and by ≥94.5% at 90 minutes; and CoCl_2_·6H_2_O suppressed ROS by ≥37.2% at 30 minutes and by ≥48.6% at 90 minutes. These results demonstrate that neither Co : NAC nor its components generated ROS; rather, they suppressed ROS production in cultured cells, suggesting that C4 would not enhance ROS production in clinical use.

## 1. Introduction

Prostate cancer is the most common type of cancer among men in the United States [1]. Low-dose-rate brachytherapy is a treatment option for men with low-risk or favorable intermediate-risk prostate cancer, providing cure rates similar to those of intensity-modulated radiation therapy [2, 3] more cost-effectively and with less severe declines in bowel health–related quality of life [4]. Oncologic outcomes and treatment-related toxicity after low-dose-rate brachytherapy depend greatly on the quality of the implant, which in turn depends on accurate placement and dose distribution of the radioactive seeds. Quality assurance efforts traditionally rely on computed tomography, which visualizes radioactive titanium seeds well but is not optimal for visualizing soft tissues such as the prostate and surrounding tissues. Magnetic resonance imaging (MRI) visualizes soft tissues well [5, 6] but requires a positive-contrast marker to precisely identify radioactive seeds [7].

Cobalt in its ionized form (Co^2+^ or Co(II)) has paramagnetic properties that generate positive-contrast signals on T1-weighted MRI. We developed an MRI marker in which cobalt chloride (CoCl_2_) is complexed with 6 water molecules (H_2_O) and the chelating antioxidant N-acetylcysteine (NAC). The resulting “cobalt complex contrast aqua solution,” or “C4,” is being tested for use in MRI-guided seed implantation in men undergoing brachytherapy for prostate cancer [7]. We have verified that the C4 MRI marker can improve visualization and localization of implanted radioactive seeds during and after implantation, and we are exploring its use to improve quality assurance and assessment of effectiveness in brachytherapy [8].

Cobalt is a naturally occurring element. In humans, a single cobalt atom is the central metal component of vitamin B_12_, a cofactor and activator of several essential enzymes that is present in most tissues, chiefly in the liver [9]. Although vitamin B_12_ is essential for erythrocyte formation, protein metabolism, and central nervous system function, cobalt and its related compounds can induce oxidative stress [10–12]. Cobalt ions have been observed to generate reactive oxygen species (ROS) in vivo and in vitro, and CoCl_2_ has been shown to induce the formation of hydroxyl radicals (^•^OH) from hydrogen peroxide (H_2_O_2_) [13]. Thus, the US Department of Health and Human Services established a minimal risk level for cobalt (0.01 mg/kg/day) [9].

The potential toxicity caused by the inadvertent release of cobalt in C4 from temporarily or permanently implanted medical devices is unknown. To reduce the potential body burden of C4 cobalt, we chelated cobalt with NAC, which is known to increase both urinary and fecal excretion of cobalt and decrease cobalt levels in the liver and spleen [14]. The purpose of the current study was to test whether the components of C4, individually or in combination, would produce reactive oxygen species including hydroxyl and peroxyl in cultured cells.

## 2. Materials and Methods

### 2.1. Cell Cultures

The human prostate cancer cell line PC3 and the human normal tongue cell line Hs-680Tg were obtained from the American Type Culture Collection (Manassas, VA, USA). The human head and neck cancer cell line HN5 was supplied by Dr. Jeffrey Myers (University of Texas MD Anderson Cancer Center, Houston, TX, USA). HN5 and Hs-680Tg cells were maintained in DMEM/F-12 medium (Corning Cellgro, Mediatech, Inc., Manassas, VA, USA). PC3 cells were maintained in RPMI-1640 medium (Sigma-Aldrich, St. Louis, MO, USA). All culture media were supplemented with 10% fetal bovine serum (FBS, Sigma-Aldrich), 100 U/mL penicillin, and 100 *μ*g/mL streptomycin (Gibco, Thermo Fisher, Grand Island, NY, USA). Cells were grown as monolayers in 75 cm^2^ flasks and maintained in a humidified 5% CO_2_/95% air atmosphere at 37°C. The identities of all cell lines were confirmed by genotyping (STR profiling) at MD Anderson's Characterized Cell Line Core Facility (NCI Core Grant CA016672).

### 2.2. Intracellular ROS Assay

Intracellular ROS levels were measured by using OxiSelect Intracellular ROS Assay Kits (Cell Biolabs, San Diego, CA, USA), which can be used to measure the activity of ROS (hydroxyl, peroxyl, and other reactive oxygen species) within a cell, according to the manufacturer's instructions. The assay uses the cell-permeable fluorogenic probe 2′,7′-dichlorodihydrofluorescein diacetate (DCFHDA), which upon diffusion into cells is deacetylated by cellular esterases to form a nonfluorescent compound that is then rapidly oxidized by ROS to create highly fluorescent 2′,7′-dichlorodihydrofluorescein (DCF). The green fluorescence intensity is proportional to the ROS levels within the cytosol [15]. The effects of antioxidants or free radical compounds on DCFHDA can be measured in terms of relative fluorescence units (RFUs). Fluorescence was measured with a VICTOR X3 multilabel plate reader (PerkinElmer, Inc., Wentzville, MO, USA) at excitation and emission wavelengths of 485 nm and 535 nm, and RFUs were quantified with PerkinElmer 2030 software.

### 2.3. Preparation of Reagents and Test Solutions

Opti-MEM (Phenol Red-negative) medium (Gibco) without FBS was used to prepare the working solution of 1000 *μ*M H_2_O_2_ (diluted from an 882 mM H_2_O_2_ stock [Thermo Fisher Scientific, Waltham, MA, USA]), the serial dilution of DCF to generate standard curves, and the 1x working solution of DCFHDA (diluted from a 20x stock supplied in the ROS Assay Kit). All the above solutions were protected from light and used immediately upon dilution. Test materials included cobalt chloride hexahydrate (CoCl_2_-6H_2_O, Fluka Sigma-Aldrich 60820, Lot 1313139) and N-acetylcysteine (NAC, Sigma-Aldrich 47250-1006, Lot 051M1820V). The ingredients were dissolved in distilled water to create weight by volume percentage (w/v%, where 1% = 1 g/100 mL) concentrations for use. Stock solutions of 10% CoCl_2_ 6H_2_O, 20% NAC, and 10% CoCl_2_·6H_2_O : 20% NAC (Co : NAC [10% : 20%]) were prepared. Final concentrations of 1% CoCl_2_·6H_2_O, 2% NAC, and Co : NAC (1% : 2%, i.e., C4) were used to treat cells in FBS-free Opti-MEM (Phenol Red-negative) medium.

### 2.4. ROS Staining and Quantification

Each cell sample was assayed in triplicate. Cells were counted with a TC 20 Automated Cell Counter (Bio-Rad Laboratories, Inc., Hercules, CA, USA). A specific number of cells (for HN5, 5 × 10^3^ cells/well; for Hs-680Tg, 8 × 10^3^ cells/well; and for PC3, 1 × 10^4^ cells/well, all cells in 100 *μ*L culture medium) were plated in clear-bottom black 96-well cell-culture plates and cultured for 24 hours. Cells were then washed with phosphate-buffered saline (PBS, Corning Cellgro) 3 times before 100 *μ*L of the 1x (for HN5 and Hs-680Tg cells) or 0.5x (for PC3 cells) DCFHDA/FBS-free Opti-MEM medium solution was added to each well. Plates were then incubated at 37°C for 30 minutes. The 1x DCFHDA/FBS-free Opti-MEM medium solution was then removed and the cells were washed 4 times with PBS. After the final wash, 90 *μ*L of FBS-free Opti-MEM medium, or 90 *μ*L of 1000 *μ*M H_2_O_2_ FBS-free Opti-MEM medium solution, was added to each well. Immediately thereafter, 10 *μ*L of the 10% CoCl_2_·6H_2_O, or the 20% NAC, or the Co : NAC (10% : 20%) was added to each well according to the different treatment conditions, and cells were incubated at 37°C. The plates were read and fluorescence was quantified before and at different times (5, 30, 60, and 90 minutes) after treatment. ROS content was reported as RFUs.

### 2.5. Cell Morphology Analysis

Each cell sample was assayed in triplicate. Cells were counted with a TC 20 Automated Cell Counter (Bio-Rad Laboratories, Inc., Hercules, CA, USA). A specific number of cells (for HN5, 5 × 10^3^ cells/well; for Hs-680Tg, 8 × 10^3^ cells/well; and for PC3, 1 × 10^4^ cells/well, all cells in 100 *μ*L culture medium) were plated in clear 96-well cell-culture plates overnight and then cells were treated with the greatest clinical exposure concentration (i.e., if 120 markers [the maximum number likely to be used in one patient with a prostate volume of 60 cc] were to leak simultaneously into the human periprostatic area after implantation) of C4 or its components (1% CoCl_2_·6H_2_O, or the 2% NAC, or the Co : NAC [1% : 2%]), and cells were incubated at 37°C. At 90 minutes after treatment, pictures of cells were taken with a bright field Evos XL core microscope (AMEX 1000, Life Technologies, Carlsbad, CA, USA) at ×10 magnification.

### 2.6. Statistical Analyses

Each experiment was repeated at least three times. Data are presented as means ± standard error of the mean (SEM). Student's *t*-tests (unpaired, unequal variance) were used to compare two groups of independent samples for ROS expression. *P* < 0.05 was considered to indicate statistical significance.

## 3. Results

### 3.1. Changes in ROS Levels in the Absence of H_2_O_2_

#### 3.1.1. Changes in ROS Levels under Control Conditions

**Figure 1:**
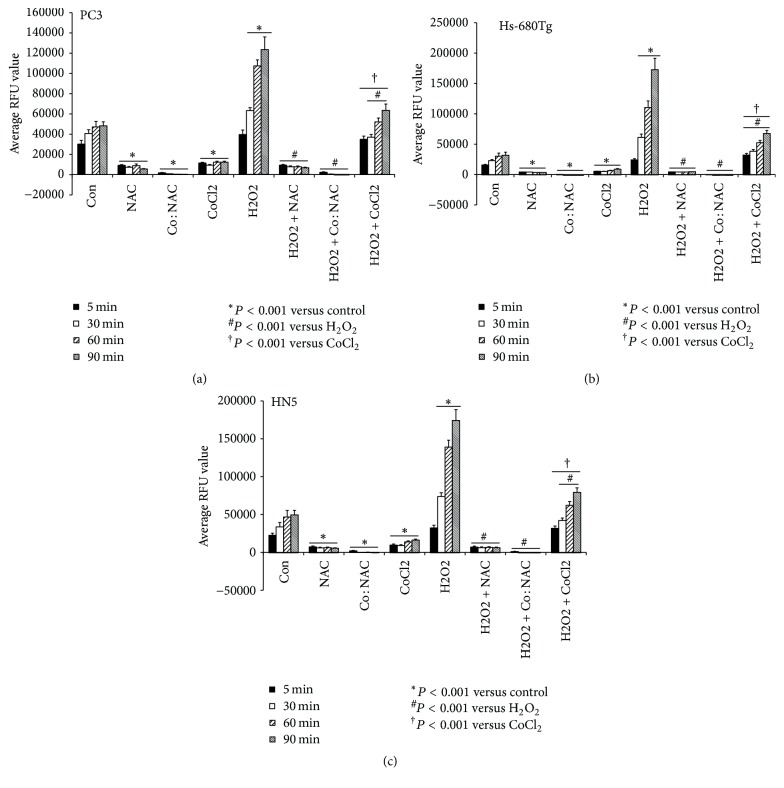
Changes in intracellular reactive oxygen species (ROS) levels over time in response to various treatment conditions. Human prostate cancer cells (PC3, (a)), human normal tongue cells (Hs-680Tg, (b)), and human head and neck cancer cells (HN5, (c)) were treated with components of the novel MRI positive-contrast marker C4 as follows: 1% [w/v] CoCl_2_·6H2O, 2% [w/v] N-acetylcysteine (NAC), or the combined Co : NAC solutions (1% : 2% [w/v]), in the presence or absence of 1000 *μ*M H_2_O_2_. At 5, 30, 60, or 90 minutes after treatment, intracellular ROS levels were evaluated. Controls (Con) are cells not treated and not exposed to H_2_O_2_. The ROS levels were measured in terms of relative fluorescence units (RFUs) at excitation and emission wavelengths of 485 nm and 535 nm. Values shown are means ± SEM from at least 3 independent experiments. Student's *t*-tests (unpaired, unequal variance) were used for comparisons.

The absolute change in RFU values for the different treatment conditions was defined as the RFUs after treatment minus the RFUs before treatment. In the absence of H_2_O_2_ or any treatment, intracellular ROS levels began to increase slightly at 5 minutes and remained high at 60 minutes (for PC3 cells, 29,893 [±4087] at 5 min versus 47,169 [±5451] at 60 min; for Hs-680Tg cells, 15,817 [±945] at 5 min versus 30,366 [±5002] at 60 min; and for HN5 cells, 22,501 [±2753] at 5 min versus 46,577 [±8787] at 60 min) and at a similar level at 90 minutes for all three cell lines (Figure 1).

#### 3.1.2. Changes in ROS Levels after Treatment with 1% CoCl_2_·6H_2_O

Compared with the untreated control condition, intracellular ROS levels in cells treated with 1% CoCl_2_·6H_2_O began to decrease as early as 5 minutes after treatment and remained at similar levels thereafter in all three cell lines (for PC3 cells, 11,329 [±930] at 5 min versus 12,404 [±935] at 90 min; for Hs-680Tg cells, 5570 [±494] at 5 min versus 8987 [±998] at 90 min; and for HN5 cells, 9645 [±1385] at 5 min versus 16,159 [±1336] at 90 min) (*P* < 0.001 versus control for all). At 5 minutes after treatment with 1% CoCl_2_·6H_2_O, the ROS levels were 37.9% of the control level in PC3 cells, 35.2% of the control level in the Hs-680Tg cells, and 42.9% of the control level in the HN5 cells (Figure 1).

#### 3.1.3. Changes in ROS Levels after Treatment with 2% NAC

Intracellular ROS levels in cells treated with 2% NAC were substantially lower than in the control condition (*P* < 0.001 for all, in all three cell lines; for PC3 cells, 9046 [±927] at 5 min versus 5571 [±446] at 90 min; for Hs-680Tg cells, 4396 [±113] at 5 min versus 3388 [±458] at 90 min; and for HN5 cells, 7238 [±1147] at 5 min versus 5487 [±604] at 90 min). At 5 minutes after treatment with 2% NAC, ROS levels were 30.3% of the control level in the PC3 cells, 27.8% of the control level in the Hs-680Tg cells, and 32.2% of the control level in the HN5 cells (Figure 1).

#### 3.1.4. Changes in ROS Levels after Treatment with Co : NAC (1% : 2%)

Treatment with Co : NAC (1% : 2%) led to very small amounts of intracellular ROS at 5 minutes after treatment (PC3 cells, 1432 [±478]; Hs-680Tg cells, 277 [±90]; and HN5 cells, 1945 [±645]); by 60 and 90 minutes, intracellular ROS levels dropped to undetectable levels in all three cell lines. At 5 min after treatment with Co : NAC, ROS levels were 4.8% of the control level for PC3 cells, 1.8% of the control level for Hs-680Tg cells, and 8.6% of the control level for HN5 cells (*P* < 0.001 versus control for all) (Figure 1).

### 3.2. Changes in ROS Levels in the Presence of H_2_O_2_

#### 3.2.1. In the Presence of H_2_O_2_ (1000 *μ*M)

Untreated cells showed a slight increase in intracellular ROS levels relative to control cells (without H_2_O_2_) at 5 minutes. Notably, ROS levels greatly increased with time in all three cell lines thereafter (at 90 minutes: for PC3 cells, 123,613 [±12,494], 255.9% of the control level; for Hs-680Tg cells, 172,750 [±18,681], 543.2% of the control level; and for HN5 cells, 174,291 [±14,274], 352.0% of the control level; *P* < 0.001 for all) (Figure 1).

#### 3.2.2. Changes in ROS Levels after Treatment with 1% CoCl_2_·6H_2_O

In the presence of H_2_O_2_ (1000 *μ*M), treatment with 1% CoCl_2_·6H_2_O led to similar intracellular ROS levels in all three of cell lines at 5 minutes compared with the no treatment group. Beginning at 30 minutes and continuing to 90 minutes, intracellular ROS levels dropped significantly relative to the untreated condition (with H_2_O_2_ only) in all three cell lines (at 90 minutes: for PC3 cells, 63,547 [±6240], 51.4% of untreated condition; for Hs-680Tg cells, 67,711 [±4988], 39.2% of untreated condition; and for HN5 cells, 79,249 [±6015], 45.5% of untreated condition; *P* < 0.05 for all). However, these values were significantly higher than those treated with 1% CoCl_2_·6H_2_O without H_2_O_2_ in all three cell lines at all 4 measurement points (for PC3 cells, 306.3% at 5 min versus 512.3% at 90 min; for Hs-680Tg cells, 574.6% at 5 min versus 753.4% at 90 min; and for HN5 cells, 330.3% at 5 min versus 490.4% at 90 min; *P* < 0.001 for all) (Figure 1).

#### 3.2.3. Changes in ROS Levels after Treatment with 2% NAC

In the presence of H_2_O_2_ (1000 *μ*M), treatment with 2% NAC led to ROS levels that were similar to those after 2% NAC without H_2_O_2_ and remained quite low over time in all three cell lines (at 90 minutes: for PC3 cells, 6774 [±617]; for Hs-680Tg cells, 4760 [±231]; and for HN5 cells, 6278 [±727]). These ROS levels were significantly lower than in the untreated condition (with H_2_O_2_ only) (for PC3 cells, 23.4% of untreated condition at 5 min versus 5.5% of untreated condition at 90 min; for Hs-680Tg cells, 18.5% of untreated condition at 5 min versus 2.8% of untreated condition at 90 min; and for HN5 cells, 21.7% of untreated condition at 5 min versus 3.6% of untreated condition at 90 min; *P* < 0.001 for all) (Figure 1).

#### 3.2.4. Changes in ROS Levels after Treatment with Co : NAC (1% : 2%)

Treatment of cells with Co : NAC (1% : 2%) in the presence of H_2_O_2_ (1000 *μ*M) led to very small amounts of intracellular ROS at 5 min (similar to levels in the Co : NAC without H_2_O_2_ condition) in all three cell lines (for PC3 cells, 2100 [±573]; for Hs-680Tg cells, 175 [±119]; and for HN5 cells, 1009 [±404]). These levels were considerably lower than the untreated condition (with H_2_O_2_ only) at 5 min (for PC3 cells, 5.3% of the untreated condition; for Hs-680Tg cells, 0.7% of the untreated condition; and for HN5 cells, 3.1% of the untreated condition; *P* < 0.001 for all) and further decreased over time to reach undetectable levels at 60 and 90 minutes in all three cell lines studied (Figure 1).

### 3.3. Cell Morphology under Treatment

**Figure 2:**
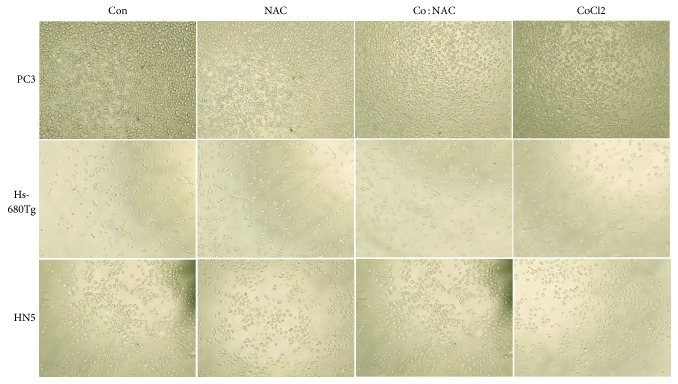
Cell morphology after various treatment conditions. Human prostate cancer cells (PC3), human normal tongue cells (Hs-680Tg), and human head and neck cancer cells (HN5) were treated with the greatest clinical exposure concentration of the novel MRI Co : NAC positive-contrast agent for C4 marker or its components (1% [w/v] CoCl_2_·6H2O, 2% [w/v] N-acetylcysteine (NAC), or the combined Co : NAC solutions (1% : 2% [w/v])). At 90 minutes after treatment, cell morphology pictures were taken with a bright field Evos XL core microscope (AMEX 1000, Life Technologies, Carlsbad, CA, USA) at ×10 magnification. Controls (Con) were untreated cells.

Compared with untreated cells, there was no histopathologic evidence of changes in cell morphology at 90 minutes after treatment with the greatest clinical exposure concentration of C4 or its components (1% CoCl_2_·6H_2_O, or the 2% NAC, or the Co : NAC [1% : 2%]) (Figure 2).

## 4. Discussion

In this study, we used a well-established ROS assay, which can be used to measure ROS (hydroxyl, peroxyl, and other reactive oxygen species) activity within a cell, to evaluate the influence of C4 and its components on the intracellular levels of ROS. The maximum tolerance concentration (1000 *μ*M) for H_2_O_2_ (i.e., that which does not lead to cell death within 24 hours) that could induce the highest levels of ROS in cells was used. We measured ROS levels over time (at 5, 30, 60, and 90 minutes after treatment) within human prostate cancer PC3 cells, human normal tongue Hs-680Tg cells, and human head and neck cancer HN5 cells after treatment with 1% CoCl_2_·6H_2_O, or 2% NAC, or the combined form Co : NAC (1% : 2%), in the presence or absence of H_2_O_2_. Standard curves were used as a quality control in these experiments. We found that one of the components of the MRI positive-contrast marker C4 (2% NAC) drastically suppressed both background ROS and the ROS in the presence of H_2_O_2_ in all three cell lines at all of the tested times (*P* < 0.001 for all). The other component of C4 (1% CoCl_2_) also significantly suppressed both background ROS levels (*P* < 0.001 for all) and the ROS in the presence of H_2_O_2_ at 30, 60, and 90 minutes after treatment in all three cell lines (*P* < 0.05 for all). Notably, the C4 marker itself (Co : NAC [1% : 2%]) drastically suppressed both basal and H_2_O_2_-related ROS levels as early as 5 minutes after treatment (*P* < 0.001 for all) and reduced ROS levels to undetectable levels at 60 and 90 minutes in all three cell lines. Moreover, no cell morphology change was observed at 90 minutes after cells were exposed to the greatest clinical exposure concentration of C4 or its components. These findings suggest that patients given C4 for seed localization during MRI-guided brachytherapy are not at risk of enhanced ROS generation from the presence of Co(II) in the C4 complex.

Ionized cobalt [Co^2+^, or Co(II)] can generate ROS [10–12] in cultured cells. ROS can induce direct cellular injury, which triggers a cascade of radical reactions enhancing secondary ROS generation. Excessive generation of ROS may further stimulate inflammatory processes involving secretion of chemotactic factors, growth factors, proteolytic enzymes, lipoxygenases, and cyclooxygenase, leading to the inactivation of antiproteolytic enzymes and the release of signaling proteins [12]. ROS is also generated by the formation of coordination complexes with metals and some chelating agents [16]. Previous reports have demonstrated that chelation of Co(II) can change its oxidation potential [17–19]. Mello-Filho and Meneghini and Sugiyama et al. investigated ^•^OH radical generation by Co(II) in the presence of chelators such as B-alanyl-3-methyl-L-histidine (anserine) and 1,10-phenanthroline and deferoxamine [20, 21]. Mao et al. reported that the extent of Co(II)-mediated generation of ROS from H_2_O_2_ depended on the properties of chelators [19], but they did not evaluate NAC. In the creation of C4, we chose NAC as the agent to block ROS from Co(II), because NAC has anti-ROS activity and can also function as an ROS inhibitor [22, 23]. In the current study, the presence of 2% NAC drastically suppressed H_2_O_2_-related ROS in HN5 cells (by up to 96.4%), Hs-680Tg cells (by up to 97.2%), and PC3 cells (by up to 94.5%) at 5–90 minutes after treatment. These findings are consistent with another report from Zou and colleagues [24]. The greater relevance to our study was their finding that treatment with NAC significantly inhibited CoCl_2_-induced apoptosis via blocking ROS production. Similarly, Jung and Kim [25] also found that NAC attenuated ROS levels induced by CoCl_2_ in the PC12 cells. Notably, when the MRI-compatible marker C4 itself (Co : NAC [1% : 2%]) was administrated, the ROS at the presence of H_2_O_2_ in HN5, Hs-680Tg, and PC3 cells was suppressed to undetectable levels starting at 30 minutes (for HN5 and Hs-680Tg cells) or at 60 minutes (PC3) after treatment. Collectively, this evidence supports the safety of the MRI marker C4 for clinical use in terms of the risk of generating ROS from interactions of Co(II) with H_2_O_2_.

In the current study, we observed to our surprise that 1% CoCl_2_·6H2O, one of the components of the MRI-compatible marker C4, could also reduce background ROS levels in all three cell lines. Even though there was H_2_O_2_-related ROS generation in the presence of 1% CoCl_2_·6H2O, those ROS levels were significantly decreased in HN5 cells (by up to 55.4%), Hs-680Tg cells (by up to 60.8%), and PC3 cells (by up to 51.5%) over time (30–90 minutes after treatment).

Our study did have limitations, since the conclusions were based on the investigation of one human prostate cancer, one human normal tongue, and one human head and neck cancer cell line under different treatment conditions. Further studies with more cell lines and in vivo models are warranted.

## 5. Conclusions

We conclude that, in human prostate cancer PC3 cells, human normal tongue Hs-680Tg cells, and human head and neck cancer HN5 cells, significant DCFH oxidation does not occur in the presence of CoCl_2_ or NAC alone or in combination; and induced (by the presence of H_2_O_2_) DCFH oxidation is blocked by CoCl_2_ and NAC and by Co : NAC. These findings provide support for the safe use of C4, in which 1% of the component CoCl_2_·6H_2_O is complexed with 2% of the chelating antioxidant NAC as a positive-contrast MRI marker for men with prostate cancer undergoing brachytherapy.
